# Proposed Amendment of the Law of Lunacy
*(1). "A Letter to the Earl of Shaftesbury on the Laws which regulate Private Lunatic Asylums," by Ed. J. Seymour, M.D., F.R.S., &c. pp. 59. Longman: 1859.(2). "Plea for the Insane Poor," by John Millar. H. Renshaw: 1859.(3). "Suggestions for the Amendment of the Laws relating to Private Lunatic Asylums," by G. Ed. T. Conolly, Barrister-at-law. Shaw and Sons: 1859.(4). "What shall we do with our Lunatics?" by Edward Eccles, F.R.C.S. J. Churchill: 1859.(5). "On some Points in the Legal Provisions for the Poor," by J. T. Arlidge, M.B. and A.B. London: 1859.


**Published:** 1859-04-01

**Authors:** 


### PROPOSED AMENDMENT OF THE LAW OF LUNACY.*

It was our intention to have discussed at a considerable length this important subject, but as the matter in all its details is at this moment under the consideration of a committee of the House of Commons, and witnesses are in the course of examination, we have thought it would be in better taste, and more consistent with the public good, to defer the publication of our remarks until the committee have completed their labours, and drawn up their report for the legislative consideration of Parliament. We cannot, however, refrain from directing the attention of our body to several valuable pamphlets that have appeared on this subject within the last few weeks. They should all be attentively read and diligently studied by every person interested in a right solution of the many difficult and important medical and social questions discussed in their pages. We would particularly refer our readers to the able essay of Dr. Seymour. Nothing can proceed from this accomplished physician's pen that is not entitled to the most

* (1). "A Letter to the Earl of Shaftesbury on the Laws which regulate Private Lunatic Asylums," by Ed. J. Seymour, M.D., F.R.S., &c. pp. 59. Longman: 1859.

(2). "Plea for the Insane Poor," by John Millar. H. Renshaw: 1859.

(3). "Suggestions for the Amendment of the Laws relating to Private Lunatic Asylums," by G. Ed. T. Conolly, Barrister-at-law. Shaw and Sons: 1859.

(4). "What shall we do with our Lunatics?" by Edward Eccles, F.R.C.S. J. Churchill: 1859.

(5). "On some Points in the Legal Provisions for the Poor," by J. T. Arlidge, M.B. and A.B. London: 1859.

careful and respectful attention. Dr. Seymour was for many years one of the Metropolitan Commissioners in Lunacy, and, therefore, he speaks as an authority on all subjects connected with the medical and domestic treatment of the insane. Many of his suggestions, as embodied in the pamphlet before us, are worthy of earnest attention. The pamphlet is written with great care, and embraces a full consideration of nearly all the points of interest connected with the subject of Lunacy Law. We beg our readers to procure the essay, and to read its interesting pages, and judge for themselves of their great merits. We again repeat that it would be ill-judged for us to quote passages from Dr. Seymour's Essay, or from any of the pamphlets alluded to, whilst the matter is still *sub judice.* We would, however, particularly call attention to the remarks of Mr. Conolly, who has evidently devoted much time and attention to the subject, as well as to the three remaining pamphlets referred to at the bottom of the page. The Committee appointed by the House of Commons has commenced its labours, and several witnesses have already been examined. The whole subject at the conclusion of the inquiry will be fully discussed in these pages.

